# The CHANGED Score—A New Tool for the Prediction of Insulin Dependency in Gestational Diabetes

**DOI:** 10.3390/jcm12227169

**Published:** 2023-11-18

**Authors:** Paul Rostin, Selina Balke, Dorota Sroka, Laura Fangmann, Petra Weid, Wolfgang Henrich, Josefine Theresia Königbauer

**Affiliations:** Department of Obstetrics, Charité—Universitätsmedizin Berlin, 10117 Berlin, Germany; selina.balke@charite.de (S.B.); dorota.sroka@charite.de (D.S.); laura.c.fangmann@gmail.com (L.F.); petra.weid@charite.de (P.W.); wolfgang.henrich@charite.de (W.H.); josefine.koenigbauer@charite.de (J.T.K.)

**Keywords:** gestational diabetes, prediction score, insulin dependency, diagnostic tool

## Abstract

Gestational diabetes (GDM) is a frequent complication during pregnancy. We aimed to develop a score to predict future insulin dependency in gestational diabetes (GDM). Data from 1611 patients from Charité Berlins gestational diabetes clinic from 2015 to 2022 were utilized. A stepwise backwards regression, including patient characteristics obtained at the initial presentation, was performed. Predictors examined included age, fasting blood glucose level, blood glucose levels one and two hours after oral glucose tolerance test, pre-pregnancy BMI, number of previous pregnancies and births, and fetal sex. The ideal cutoff value between high and low risk for insulin dependency was assessed and the score was internally validated. There were 1249 (77.5%) women diagnosed with dietary GDM and 362 (22.5%) were diagnosed with insulin-dependent GDM. The CHarité AssessmeNt of GEstational Diabetes (CHANGED) Score achieved an area under the curve of 0.77 (95% confidence interval 0.75–0.80; 0.75 in internal validation). The optimal cutoff value was calculated at a score value of 9 (72% sensitivity, 69% specificity). We developed an easily applicable tool to accurately predict insulin dependency in gestational diabetes. The CHANGED Score is routinely available and can potentially improve maternal and fetal outcomes.

## 1. Introduction

Gestational diabetes mellitus (GDM) is a condition defined as a glucose intolerance first recognized during pregnancy that is not overt diabetes [1]. It represents the most common metabolic disorder and medical complication during the course of pregnancy and is estimated to effect two to nine percent of pregnancies worldwide; when employing the new International Association of Diabetes and Pregnancy Study Groups (IADPSG) criteria, GDM’s worldwide prevalence may be as high as 18 percent [2,3,4,5].

The rate of GDM in Germany rose from 4.6% of all hospital deliveries in 2013 to 6.8% of deliveries in 2018 [6]. This trend can also be detected in other western countries, and may correlate with an increased obesity rate as well as a higher average age at conception [7,8]. The treatment of GDM may thereby continue to become a more and more acute public-health challenge. GDM typically manifests as a transient condition that resolves after childbirth; however, it constitutes a major risk factor for the future development of type 2 Diabetes, with some studies estimating a risk as high as 30% after GDM [9]. GDM is further associated with various perinatal complications for the mother and neonate (e.g., preeclampsia, spontaneous abortion, fetal anomalies and macrosomia, cesarean delivery, and neonatal hypoglycemia) [6,10,11,12,13,14].

The extent of its effect on maternofetal health is primarily determined by the severity of the disease, which can generally be described as either moderate (dietary GDM) or severe (insulin-dependent GDM) [15,16]. The distinction between the two progression types of GDM is essential, not only to be able to provide the correct treatment, but also in order to choose the correct patient-management strategy (i.e., frequency of appointments, intervals of escalation, interventional therapy, planning of delivery).

Adequate monitoring and treatment of gestational diabetes can improve the perinatal outcomes. An intensified management and follow-up of patients with insulin-dependent GDM (iGDM) has been, along with other societies, suggested by the American College of Obstetricians and Gynecologists [17,18,19]. However, it can be difficult to foresee the course of disease based on the first consultation and the oral glucose tolerance tests performed at this point. Recent investigations have characterized different manifestations of disease, or so-called metabolic types, depending on the response to the 75 g oral glucose tolerance test (oGTT) [20,21]. It may be possible to utilize these findings by including oGTT patterns, as well as known risk factors, into a new prediction model that clinicians can apply to improve the identification at an early point of pregnancy of patients at risk of iGDM, thereby being able to optimize and individualize treatment plans.

## 2. Materials and Methods

**Figure 1:**
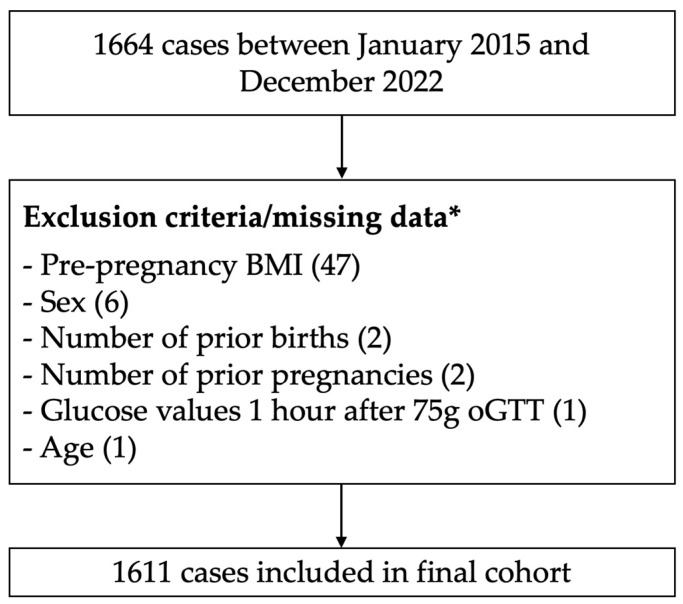
Study flow. * One criterion can apply to multiple patients/cases.

In this study, we utilized a database of the Charité gestational diabetes clinic from January 2015 to December 2022. The Charité University Hospital is a large perinatal center in Berlin, Germany. The core dataset had already been used for another investigation by our group [22]. We included 1664 patients over the age of 18 with singleton pregnancies, pathological glucose response in the 75 g oGTT screening, and subsequent delivery at Charité Berlin. We diagnosed GDM and initiated insulin therapy based on the International Association of Diabetes and Pregnancy Study Groups (IADPSG) criteria and excluded women with missing information for predictor variables (Figure 1) [23].

Fasting glucose and values 1 h and 2 h after oGTT, as well as the age and BMI before conception, were categorized into quintiles; the numbers of pregnancies and births were divided into two groups along their respective medians. Fetal sex was classified as a binary variable.

Metformin was not routinely utilized at our institution over the course of this study.

After categorization, we performed a stepwise backwards logistic regression with a cutoff for significance of *p* < 0.05 to identify potential predictors for insulin-dependent GDM as well as their respective weights. In accordance with the German gestational diabetes guidelines, insulin therapy at our institution was initiated in women who presented with elevated blood glucose levels in ≥50%, repeated fasting glucose of ≥110 mg/dL, or in cases of fetal macrosomia with a disproportionate head circumference to abdominal circumference ratio [24].

The weights assigned to individual predictors in the stepwise backwards logistic regression analysis were transformed to the score values by setting the smallest weight to 1 and dividing all other weights by the original smallest weights value.

The model discrimination of the CHarité AssessmeNt of GEstational Diabetes Score was evaluated by performing C-statistics. The model reliability, calibration, and resolution were assessed by Brier score calculation (squared difference between predictions and actual event rates) [25]. The Hosmer–Lemeshow test was performed to investigate the goodness-of-fit of the model (a *p* value > 0.05 indicating good model fit) [26].

The optimal cutoff value to differentiate between high and low risk for insulin-dependent GDM was determined, based on the Liu Index, optimizing sensitivity and specificity [27]. A calibration plot was created to visualize the range of score values and risks encompassed by the CHANGED Score.

We internally validated the score, utilizing ten-fold cross-validation (10 iterations of score generation leaving out 1/10th of the cohort each time and performing validation on the remaining 1/10th) [28].

The ethics review committee of Charité Berlin granted its approval on 6th February 2023 (EA2/255/22). Statistical analyses were performed using Stata 13 (Stata Corp LLC, College Station, TX, USA).

## 3. Results

After the exclusion of 53 women due to missing information on age, 75 g oGTT 1-h values, BMI before conception, or number of prior pregnancies and births, the final cohort consisted of 1611 patients (Figure 1).

There were 1249 (77.5%) patients diagnosed with dietary GDM and 362 (22.5%) with insulin-dependent GDM. The cohort characteristics are displayed in Table 1.

All considered predictors except for “number of previous births” met the cutoff for significance in one or more quintiles/categories: Male sex (vs. female) coefficient 0.37, Odds Ratio (OR) 1.44, *p* = 0.007, fasting glucose 90–94 mg/dL (vs. <90 mg/dL) coefficient 0.83, OR 2.30, *p* = 0.002, fasting glucose 95–98 mg/dL (vs. <90 mg/dL) coefficient 1.02, OR 2.78, *p* < 0.001, fasting glucose 99–106 mg/dL (vs. <90 mg/dL) coefficient 1.52, OR 4.54, *p* < 0.001, fasting glucose >106 mg/dL (vs. <90 mg/dL) coefficient 2.14, OR 8.50, *p* < 0.001, 3 or fewer pregnancies (including the current one) coefficient 0.32, OR 1.38, *p* = 0.028, BMI before pregnancy 26.2–29.3 (vs. BMI ≤ 23 kg/m^2^) coefficient 0.62, OR 1.85, *p* = 0.002, BMI before pregnancy 29.4–33.2 (vs. BMI ≤ 23 kg/m^2^) coefficient 1.10, OR 3.01, *p* < 0.001, BMI before pregnancy >33.2 (vs. BMI ≤ 23 kg/m^2^) coefficient 1.26, OR 3.50, *p* < 0.001, 1 h oGTT > 208 mg/dL (vs. ≤150 mg/dL) coefficient 0.51, OR 1.68, *p* = 0.002, 2 h OGTT > 167 mg/dL (vs. <114 mg/dL) coefficient 0.41, OR 1.45, *p* = 0.032, age 29–31 (vs. <29) coefficient 0.68, OR 1.97, *p* = 0.002, age 32–34 (vs. <29) coefficient 0.61, OR 1.84, *p* = 0.003, age 35–37 (vs. <29) coefficient 0.86, OR 2.33, *p* < 0.001, age > 37 (vs. <29) coefficient 0.67, OR 1.96, *p* = 0.002.

**Figure 2:**
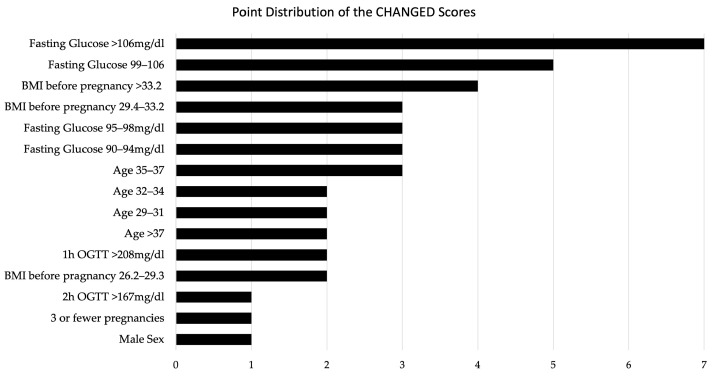
Point Distribution of the CHANGED Scores.

The respective score values for each individual predictor are displayed in Figure 2.

**Figure 3:**
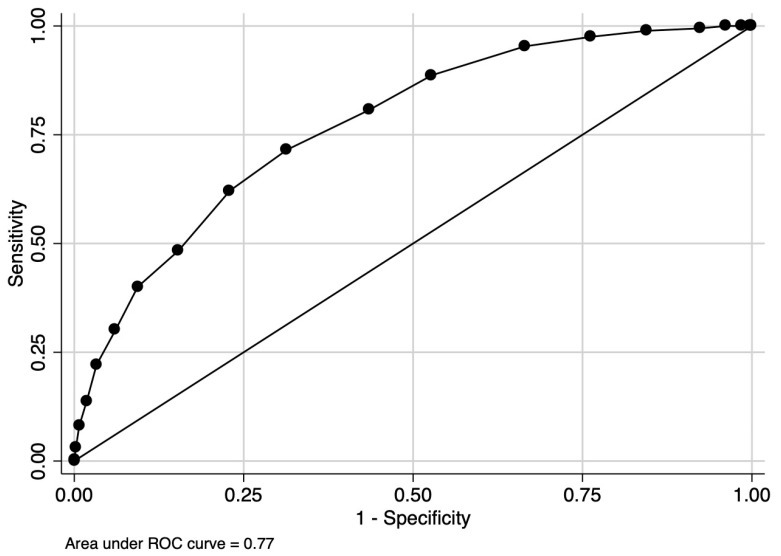
AUC of the CHANGED Score.

**Figure 4:**
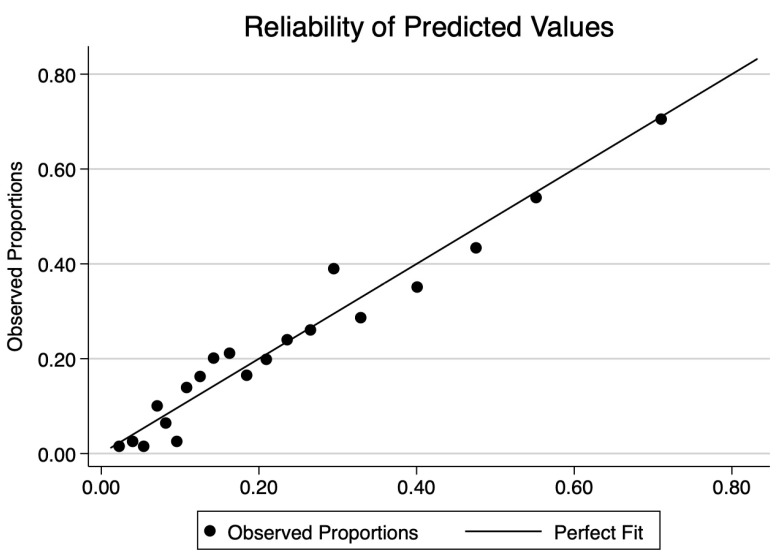
Reliability plot of the CHANGED Score.

**Figure 5:**
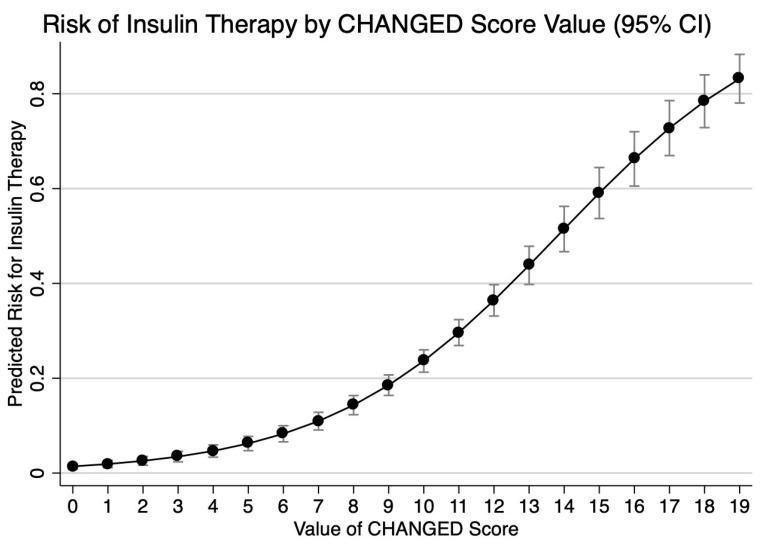
Risks across CHANGED Score values; 1 = 100%.

The final CHarité AssessmeNt of GEstational Diabetes Score yielded an area under the curve (AUC) of 0.77 (95% CI 0.75–0.80) (Figure 3). Hosmer–Lemeshow goodness-of-fit testing (*p* = 0.18), a Brier score of 0.14, and the well-aligned reliability graph (Figure 4) demonstrated good model fit, resolution, calibration, and reliability across risk groups. The CHANGED Score encompassed a wide range of score values and risks (0 to 19; 14% to 83% risk) (Figure 5). A CHANGED Score of above 15 corresponded to a risk of >60% for insulin-dependent GDM, while a point value under 10 corresponded to less than a 20% risk. The optimal cutoff value (Liu Index) between high and low risk for insulin-dependent GDM was at a score value of 9 (72% sensitivity, 69% specificity, AUC 0.70). This cutoff value possessed a positive predictive value of 0.40 and a negative predictive value of 0.90.

Ten-fold cross-validation yielded an average AUC of 0.75 (minimum of 0.69, maximum of 0.83).

## 4. Discussion

### 4.1. Main Findings

The CHANGED Score achieved good predictive accuracy for identifying insulin-dependent GDM (AUC 0.77). The optimal cutoff value for differentiating between high and low risk for iGDM was 9, with a sensitivity of 72%, and a specificity of 69%. With a negative predictive value of 0.9—indicating that 90% of patients who were assessed to not develop iGDM did in fact not develop insulin dependency—it is well suited to help in determining which patients are best served with longer intervals between checks.

The highest score value for individual predictors was obtained by elevated fasting blood glucose levels > 106 mg/dL with 7 points. A high pre-pregnancy BMI was also identified as an important predictor of insulin dependency with 4 points assigned for BMI > 33.2. The risk for iGDM also increased with age up to the age of 37, with 2 points assigned to the age group between 29 and 34, and 3 points given to the group between 35 and 37. Being older than 37, however, correlated with a lower risk than a maternal age between 35–37 (2 points assigned). This finding may be indicative of the influence of lifestyle factors among women who become pregnant after the age of 37 (potentially higher socioeconomic status, healthier diet, history fertility treatment etc.). The exact factors associated remain to be investigated. A male sex of the fetus and having three or fewer previous pregnancies were both assigned a score of 1 point in the predictive model. Moreover, elevated blood glucose levels during the oral glucose tolerance test, particularly at the one-hour mark, were identified as significant predictors of insulin dependency, resulting in a score of 2 points for > 208 mg/dL. Score values for each predictor are presented in Figure 2.

### 4.2. Results in Context of the Literature

Our results are consistent with previous studies that have reported associations between these individual factors and the severity of GDM. For example, advanced maternal age or other specific findings have been consistently identified as risk factors for GDM progression and insulin dependency [22,29]. Similarly, elevated fasting blood glucose levels and impaired glucose tolerance during the oral glucose tolerance test have been linked to an increased likelihood of insulin therapy [30]. Additionally, pre-pregnancy BMI has been established as an important predictor of GDM severity and treatment requirements [31,32].

Although these risk factors have been identified and studied thus far, their comprehensive application has been limited in routine clinical practice. A scoring system provides a path to weigh different risk factors and enable clinicians to provide individual, targeted guidance and therapy. Previous models, such as that developed by Lee et al., have aimed to stratify GDM patients based on their risk for insulin therapy based on regression models as well [33].

In addition to readily available variables, such as age, BMI, and fasting glucose—factors that were also utilized in this study—Seung-Hwan Lee et al. incorporated total cholesterol and γ-glutamyl transferase levels as risk factors. These variables are not routinely available, and clinicians would need to test for them solely to be used in the prediction model. The availability and potential extra costs of additional factors constitute important limitations of Seung-Hwan Lee et al.’s investigation, and may be a major strength of our study. Another difference is the overall purpose: Our study focused specifically on pregnant women already diagnosed with GDM, whereas Seung-Hwan Lee et al.’s study aimed to predict insulin-requiring GDM before pregnancy. Both studies achieved comparable AUC values, with AUC of 0.783/0.77. Their findings highlight the significance of a woman’s health status before her pregnancy in relation to the development of gestational diabetes.

Another prediction model was developed by Pertot et al., who conducted a prospective study analyzing data from 3009 GDM patients at the Royal Prince Alfred Hospital clinic in Australia to identify risk factors for insulin treatment, between 1995 and 2010 [20]. The authors found ethnicity, gestation at the time of diagnosis, HbA1c, glucose levels in oGTT, BMI, and a family history of diabetes to predict iGDM. However, their model only reached a sensitivity of 66% and a specificity of 37% (compared to our model’s sensitivity of 72% and specificity of 69% at the cutoff of 9). It is worth noting that the Pertot et al. study reported a higher rate of insulin therapy, with 51% of women requiring treatment, whereas a rate of only 22.5% was found in our cohort. This discrepancy significantly impacted the predictive value of each factor, underlining the differences in diagnosing varying populations and making it harder to build a risk engine with high specificity in Pertot et al.’s cohort.

Despite these variations, both studies highlight the challenges associated with predicting insulin therapy in patients with GDM solely based on clinical and biochemical characteristics at the time of diagnosis. Consequently, there is a need to re-evaluate the risk-scoring system in different population groups to enhance its accuracy.

Another well-crafted prediction model was designed by Eleftheriades et al. utilizing Classification and Regression Trees, which reached a similar AUC compared to our model (0.74 vs. 0.75 in our model in internal validation) [19]. Another similarity was the share of iGDM in the cohort (17% vs. 23%). While our study confirmed most of the found prediction factors and their weights (baseline glucose levels, oGTT levels, maternal age) differences can be found in cohort size (775 in Eleftheriades et al. vs. 1611 in this study), variable classification (more continuous variables in Eleftheriades et al.), and potential application.

While the aforementioned work described and calculated the interplay of various risk factors, this study provides the reader with a clear-cut, point-based model as well as cutoff values that can easily be implemented in clinical practice. Only the implantation of such models can contribute to the improvement of outcomes. Our group plans to provide accessible online tools to assist with the implementation after external, prospective validation of this study. Applying a scoring system and informing patients of their increased risk for iGDM, especially if non-compliant, may already serve as a communicative tool to increase compliance overall [19].

Furthermore, as insulin treatment is also associated with postpartum dysglycemia, identification of patients through application of the CHANGED Score may also enable an intensified postpartum follow-up and may continue to increase the compliance after childbirth [34].

It is essential to identify women in need of GDM treatment in pregnancy as swiftly as possible in order to mitigate potential maternal and fetal complications [35]. A randomized controlled trail recently published in the New England Medical Journal by Simmons et al. found improved neonatal outcomes when GDM/iGDM is treated before 20 weeks of gestation, potentially indicating a linear increase of risk the longer undesirable glucose levels remain without treatment and the more severely they diverge from the recommended range [36].

In 2005, Crowther et al. presented a large randomized clinical trial finding that targeted treatment of GDM (including insulin), as opposed to routine care at the time, reduced composite serious perinatal complications (defined as defined as death, shoulder dystocia, bone fracture, and nerve palsy) from 4 percent to 1 percent [37].

More recently, in 2020, Harrison et al. retrospectively evaluated a more rigorous threshold for the start of pharmacotherapy (insulin or oral hypoglycemic agent) in women with GDM (start when 20–39% of capillary blood glucose values are above goal instead of when ≥40% capillary blood glucose values are above goal) [38]. They found that the application of the lower threshold (20–39% of capillary blood glucose values above goal) was associated with decreased rates of complication (composite outcome consisting of macrosomia, large for gestational age fetus, shoulder dystocia, hypoglycemia, hyperbilirubinemia requiring phototherapy, respiratory distress syndrome, stillbirth, and neonatal demise). Analyzed individually, Harrison et al. observed NICU admission and LGA rates to be higher in the less restrictive group (pharmacotherapy when ≥40% capillary blood glucose values are above goal).

Tartaglione et al. recently published an investigation utilizing continuous glucose monitoring in pregnant women with normal oGTT. Of 53 women with normal oGTT, they found 33 women to display abnormal glycemic patterns, 12 of whom were requiring insulin, suggesting that the number of women affected by GDM may be even higher and risk and GDM prediction models might find an even more general application in the future [39].

Based on these findings, we hypothesize that early and consistent identification of iGDM through employment of the CHANGED score may lead to earlier treatment and improved maternofetal outcomes.

Risk stratification tools such as the CHANGED Score are also needed so patients identified as “high risk” can undergo short-interval checks, while those at low risk can be seen at longer intervals, thereby potentially reducing healthcare costs. Further investigations are required in order to substantiate or disprove these hypotheses.

### 4.3. Strengths and Limitations

The study utilized a large sample size with data from 1611 patients from Charité Berlin’s gestational diabetes clinic. Our database recorded routinely collected data, which eliminates the need for additional testing and facilitates its practical implementation in routine clinical procedures. This characteristic enhances the usability and feasibility of the scale in real-world healthcare settings. It is important to note that this study has several limitations. The study was conducted at a single center, limiting the generalizability of the findings to other healthcare settings or populations. Selection bias may have been present, as the study included patients from a specific clinic, potentially affecting the representativeness of the patient population. The developed scoring system has not been validated in an independent cohort, which may impact its generalizability and reliability in different populations.

## 5. Conclusions

The CHarité AssessmeNt of GEstational Diabetes (CHANGED) Score contributes to the understanding of the risk factors for insulin dependency in gestational diabetes patients.

It achieved high predictive accuracy (AUC 0.77), was well calibrated (sensitivity of 72% and specificity of 69% at cutoff value of 9), and maintained its reliability in an internal validation (average AUC 0.75).

Healthcare professionals can utilize this new prediction tool to identify patients who are at a higher risk of developing insulin dependency and tailor their management and treatment plans accordingly to optimize maternal and fetal outcomes in gestational diabetes cases.

The CHANGED score is easy to implement and can potentially improve maternal and fetal outcomes. Further studies are necessary to analyze the effects of its clinical implementation and externally validate this new instrument in diverse populations.

## Figures and Tables

**Table 1:** Study cohort characteristics.

Characteristics	No Insulin (n = 1249)	Insulin (n = 362)	*p*-Value
Sex, female	580 (46.4%)	141 (39.0%)	0.012
Age, median (IQR)	32.00 (28.00, 36.00)	34.00 (30.00, 37.00)	0.001
BMI_early, median (IQR)	26.60 (23.10, 31.10)	30.50 (27.20, 35.30)	<0.001
Glucose_0 h, median (IQR)	95.00 (89.00, 101.00)	103.00 (96.00, 111.00)	<0.001
Glucose_1 h, median (IQR)	181.00 (155.00, 198.00)	193.00 (169.00, 219.00)	<0.001
Glucose_2 h, median (IQR)	136.00 (116.00, 158.00)	149.00 (126.00, 176.00)	<0.001
Parity, median (IQR)	1.00 (0.00, 2.00)	1.00 (0.00, 2.00)	0.002
Gravida, median (IQR)	3.00 (2.00, 4.00)	3.00 (2.00, 4.00)	0.008

## Data Availability

The data presented in this study are available on request from the corresponding author.
